# The Effects of Nutrition on the Gastrointestinal Microbiome of Cats and Dogs: Impact on Health and Disease

**DOI:** 10.3389/fmicb.2020.01266

**Published:** 2020-06-25

**Authors:** Susan M. Wernimont, Jennifer Radosevich, Matthew I. Jackson, Eden Ephraim, Dayakar V. Badri, Jennifer M. MacLeay, Dennis E. Jewell, Jan S. Suchodolski

**Affiliations:** ^1^Hill’s Pet Nutrition, Inc., Topeka, KS, United States; ^2^Department of Grain Science and Industry, Kansas State University, Manhattan, KS, United States; ^3^Texas A&M College of Veterinary Medicine & Biomedical Sciences, College Station, TX, United States

**Keywords:** microbiome, cats, dogs, metabolism, nutrition, macronutrient, prebiotic, postbiotic

## Abstract

The gastrointestinal (GI) microbiome of cats and dogs is increasingly recognized as a metabolically active organ inextricably linked to pet health. Food serves as a substrate for the GI microbiome of cats and dogs and plays a significant role in defining the composition and metabolism of the GI microbiome. The microbiome, in turn, facilitates the host’s nutrient digestion and the production of postbiotics, which are bacterially derived compounds that can influence pet health. Consequently, pet owners have a role in shaping the microbiome of cats and dogs through the food they choose to provide. Yet, a clear understanding of the impact these food choices have on the microbiome, and thus on the overall health of the pet, is lacking. Pet foods are formulated to contain the typical nutritional building blocks of carbohydrates, proteins, and fats, but increasingly include microbiome-targeted ingredients, such as prebiotics and probiotics. Each of these categories, as well as their relative proportions in food, can affect the composition and/or function of the microbiome. Accumulating evidence suggests that dietary components may impact not only GI disease, but also allergies, oral health, weight management, diabetes, and kidney disease through changes in the GI microbiome. Until recently, the focus of microbiome research was to characterize alterations in microbiome composition in disease states, while less research effort has been devoted to understanding how changes in nutrition can influence pet health by modifying the microbiome function. This review summarizes the impact of pet food nutritional components on the composition and function of the microbiome and examines evidence for the role of nutrition in impacting host health through the microbiome in a variety of disease states. Understanding how nutrition can modulate GI microbiome composition and function may reveal new avenues for enhancing the health and resilience of cats and dogs.

## Introduction

The word “microbiome” has been traditionally defined as “the aggregate genetic material of all microorganisms living in, or on, a defined habitat” (Lederberg and McCray, 2001; The NIH HMP Working Group, 2009). More generally, the term “microbiome” now refers to both the bacterial cells themselves and their genetic material. Given the wide ranging and often profound effects of the microbiome on the health of both humans and pets, the gastrointestinal (GI) microbiome is now recognized as an organ (Possemiers et al., 2011) with unique metabolic capabilities. The GI microbiome is comprised of trillions of cells residing in the digestive tract, which begins in the oral cavity and continues to the rectum (Gibson and Roberfroid, 1995). While the bacterial component of the microbiome has been the target of much research and represents the focus of this review, the microbiome also consists of fungi, archaea, viruses, and protozoa.

**FIGURE 1:**
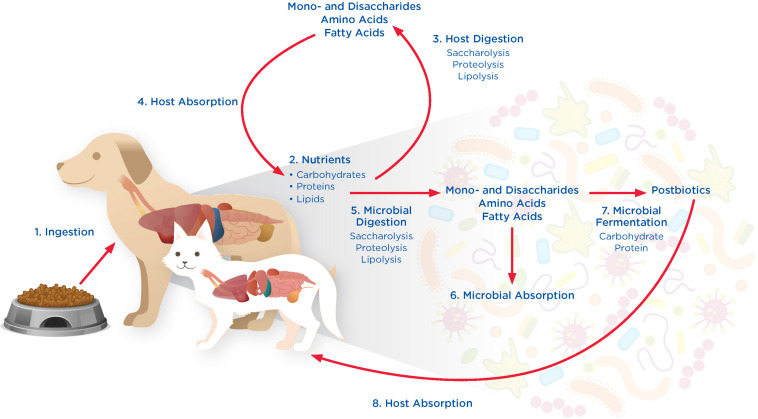
Tripartite interactions between pet foods, the GI microbiome, and host in 8 phases. **(1) Ingestion:** Dogs and cats ingest nutrients such as carbohydrates, protein, and lipids, in the form of pet foods that are provided to them. **(2) Nutrients:** Nutrients from pet foods enter the GI tract where they are available for digestion by the host and microbiome. **(3) Host Digestion:** Digestion by the host involves processes such as saccharolysis, proteolysis, and lipolysis, releasing mono- and disaccharides, amino acids, and fatty acids. **(4) Host Absorption:** Mono- and disaccharides, amino acids, and fatty acids produced through host digestion can be absorbed and utilized by the host cells. **(5) Microbial Digestion:** Nutrients not digested or absorbed by the host are available for digestion by the microbiome through saccharolysis, proteolysis, and lipolysis, releasing mono- and disaccharides, amino acids, and fatty acids. **(6) Microbial Absorption:** Mono- and disaccharides, amino acids, and fatty acids generated through microbial digestion can be absorbed and utilized by the microbiome. **(7) Microbial Fermentation:** Nutrients in excess of host and microbe absorptive capabilities are bypassed to the lower GI tract where they can undergo microbial fermentation to produce postbiotics that can impact host health locally within the GI tract. **(8) Host Absorption:** Microbe-derived postbiotics can also be absorbed by the host, impacting host health at locations outside of the GI tract.

Development of the microbiome begins before birth (Stinson et al., 2019) and the microbiome influences many aspects of host health, including physiology, anatomy, behavior, reproduction, and fitness (Bordenstein and Theis, 2015; Thomas et al., 2017). For example, the GI microbiome facilitates the breakdown of food (Figure 1) as well as the production of metabolites, such as short chain fatty acids (SCFAs), secondary bile acids, vitamins (D’Argenio and Salvatore, 2015; Suchodolski, 2016), nutrients, and other bacterially derived compounds (Mondo et al., 2019). The microbiota release nutrients and metabolites into the body, influencing immune cells and inflammatory functions (Tizard and Jones, 2017). In return, the host provides an infrastructure for the microbiome, regulates its temperature and oxygen levels, controls peristalsis, provides pathogen defense, and offers the anatomical scaffolding and structure that serves as the environmental habitat for the GI microbiome. A mucus-lined epithelial barrier serves as the interface between the host and resident microbes, in addition to promoting immune surveillance and facilitating host-microbiome homeostasis. Evidence also suggests that the GI microbiome influences the development and regulation of major host systems, including the nervous, renal, digestive, dermal, endocrine, immune, and respiratory systems (Evenepoel et al., 2017; Tizard and Jones, 2017; Makki et al., 2018). For example, commensal bacteria play a key role in the function of the host immune system, which is essential for the development of the physiologic structure of the gut (Mondo et al., 2019).

The composition of the microbiome across the GI tract is not uniform, as qualitatively and quantitatively distinct communities have been identified in each ecological and anatomical niche (Alarcón et al., 2017; Proctor and Relman, 2017; Gorkiewicz and Moschen, 2018; Yadav et al., 2018). Research in pets and humans now recognizes distinct microbiomes in the oral cavity (Dewhirst et al., 2012, 2015; Davis, 2016), esophagus (Corning et al., 2018), and stomach (Bik et al., 2006; Gorkiewicz and Moschen, 2018). Even within a single anatomical region, evidence in humans and dogs suggests there may be distinct subpopulations that vary with the terrain and topography of the region, as well pH, oxygen, and nutrient gradients (Suchodolski et al., 2008; Segata et al., 2012; Wlodarska et al., 2015; Hoffmann et al., 2016; Honneffer et al., 2017; Proctor and Relman, 2017; Gorkiewicz and Moschen, 2018). In addition, the biogeography of bacteria within the gut is shaped by several factors, including diet, antimicrobials, mucus, and the host immune system (Donaldson et al., 2016).

The community of microbes across the GI tract differ, representing the microenvironment and physiologic functions of each intestinal segment (Pilla and Suchodolski, 2020) and this has been demonstrated both in dogs and in humans. For example, two studies of healthy domestic dogs found significant differences between the microbiome of the small and large intestines, including greater representation of *Proteobacteria* in the duodenum versus colon or rectum, and increases in *Lachnospiraceae* and *Ruminococcaceae* in the large intestine versus small intestine (Suchodolski et al., 2008; Honneffer et al., 2017). One of these studies also characterized the metabolome, finding greater concentrations of amino acids and pyruvate in the small intestine and higher concentrations of phenol-containing carboxylic acid compounds in the large intestine (Honneffer et al., 2017). In general, the largest, most complex, and best studied microbiome in the GI tract is located in the colon. Compared with the microbiome of the large intestine, the small intestine microbiome appears to be more sensitive to dietary changes and in humans, carbohydrate fermenting species belonging to *Streptococcus* and *Veillonella* genera are regularly encountered (El Aidy et al., 2015). The small intestine is populated with mostly aerobes and facultative anaerobes, while the primary populations in the cecum and the descending colon are facultative anaerobes and strict anaerobes, respectively, corresponding to the decreasing oxygen gradient along the GI tract (Yadav et al., 2018; Pilla and Suchodolski, 2020). It has been proposed that the small intestinal microbiota is oriented around utilization of simple, diet-derived carbohydrates (El Aidy et al., 2015). Since microbiota samples are difficult to obtain from the GI tract, studies typically utilize fecal samples. It has been suggested that canine fecal samples provide better representation of bacterial taxa relevant to health compared to human samples, which may be due to the fact that dogs have a shorter GI tract and more rapid transit time with fewer mucusa-associated taxa than humans (Vázquez-Baeza et al., 2016; Pilla and Suchodolski, 2020). It has also been shown that the predominant bacterial phyla of the gut microbiome in healthy dogs—*Bacteroidetes*, *Firmicutes*, *Proteobacteria*, and *Fusobacteria*—is similar to that in humans (Swanson et al., 2011). Yet, other analyses have found that despite some similar changes in bacterial taxa, the gut microbiome of dogs with IBD is largely distinct from that of humans with IBD (Vázquez-Baeza et al., 2016).

In contrast to humans, cats and dogs do not rely on the microbiota for energy (Deng and Swanson, 2015). Domestic cats require foods with high protein content to meet their nutritional needs and use smaller amounts of glucose (Deng and Swanson, 2015). While dogs have many of the same anatomical and metabolic traits as cats, they are typically more omnivorous and can digest, absorb and metabolize much higher amounts of dietary carbohydrates (Deng and Swanson, 2015).

In general, the composition of the gut microbiota is similar between cats and dogs (Hoffmann et al., 2016). *Firmicutes*, *Bacteroidetes*, *Proteobacteria*, *Fusobacteria*, and *Actinobacteria* are reported to be the dominant microbial phyla in the gut for both cats and dogs (Deng and Swanson, 2015; Hoffmann et al., 2016); however, several studies have noted differences between the two species (Handl et al., 2011; Jha et al., 2020). While a recent study comparing the gut microbiomes of 46 cats and 192 dogs based on fecal samples yielded results that were relatively consistent with those stated above, it also revealed that cats exhibited higher alpha diversity than dogs. Compared to cats, bacterial phyla elevated in dogs were *Enterococcu*s, *Fusobacterium*, *Megamonas* and *SMB53*, while multiple phyla were more adundant in cats, including *Adlercreutzia*, *Alistipes*, *Bifidobacterium*, *Carnobacterium*, *Collinsella*, *Coprococcus*, *Desulfovibrio, Faecalibacterium*, *Oscillospira*, *Parabacteroide*s, *Peptococcus*, *Peptostreptococcus*, *Ruminococcus*, *Slackia*, and *Sutterella* (Jha et al., 2020). A more diverse GI microbiome among cats versus dogs was also observed in an earlier analysis, based on a higher numer of operational taxonomic units (113 in cats versus 85 in dogs); however, this same study also revealed fewer interindividual differences in the abundance of most bacteria in cats and a greater number of cats with the same bacterial genera (Handl et al., 2011). Differences between cats and dogs were also apparent in the fungal microbiome as *Nakaseomyces* predominated in dogs, while *Saccharomyces*, *Aspergillus*, and *Penicillium* were more abundant in cats. Such differences may be due to adaptation of the microbiome to different diets (i.e., greater interindividual diversity among dogs may be a product of a more varied, omnivorous diet compared with the carnivorous diet of cats) (Handl et al., 2011).

The proportions of phyla can also vary among individual animals of the same species due to factors such as breed, diet, age, living environment, and differing methods of analysis across studies (Deng and Swanson, 2015; Jha et al., 2020; Pilla and Suchodolski, 2020). However, most studies compared only small numbers of animals and larger cohort studies are neeed to confirm the presence of environmental effects and estimate effect sizes.

There is also evidence that members of microbiomes from other portions of the GI tract are important reservoirs for the communities found in the intestine. For example, members of the oral microbiome have been found to colonize lower portions of the GI tract where they have been associated with disease (Lira-Junior and Bostrom, 2018).

Given the potential impact of the GI microbiome on pet health, both directly and through its impact on the response to nutrition (Suez and Elinav, 2017), as well as an increasing awareness of the diagnostic and therapeutic potential of interventions targeting the GI microbiome, the objectives of this review are to: (1) discuss metrics used to evaluate the microbiomes of cats and dogs; (2) describe the impact of nutritional interventions on the microbiome of cats and dogs; (3) consider current evidence for the role of nutrition in influencing cat and dog health through changes to the GI microbiome; and (4) explore emerging evidence for the role of microbiome function and postbiotic effects on cat and dog health.

## Metrics to Evaluate the Microbiome of Cats and Dogs

To evaluate research on the microbiome, it is important to first consider how microbiome status is assessed. A variety of metrics have been used in the literature to characterize microbiome status, including measurements representing both compositional and functional characteristics of the microbiome (Backhed et al., 2012). Most published microbiome studies involving cats and dogs use measurements of microbial composition rather than function when describing the microbiome or assessing the relationship between the microbiome and host health. Such metrics of microbiome composition may include relative abundance of various microbial taxa, as well as assessments of dysbiosis, which has been proposed to describe “an altered composition of the commensal microbiota that is detrimental to the host” (Staley et al., 2018). In addition, measurements of alpha or beta diversity, which reflect the richness and/or evenness of the distribution of bacterial groups within and between bacterial communities, respectively, have also been used (Staley et al., 2018). While common, measurements of microbial composition have limitations, including a higher degree of variation between individuals than over time within an individual (Schloissnig et al., 2013; Franzosa et al., 2015; Lloyd-Price et al., 2017); this high degree of interindividual variation complicates the identification of microbiome compositions that can reliably characterize health or disease and contributes to limited reproducibility across populations (Schloss, 2018). Indeed, factors that vary among (and within) individuals such as diet, environment, medication use, developmental stage, genetics, and health status have been shown to influence microbiome composition to some degree (Goodrich et al., 2016; Doestzada et al., 2018; Makki et al., 2018; Yadav et al., 2018), and even within healthy individuals, microbiome compositions have been shown to vary widely and continuously over time (Knights et al., 2014).

The health of the microbiome may be more meaningfully defined by how it functions, rather than its taxonomic or compositional makeup. Functional redundancy has been identified in multiple taxa from site-specific bacterial communities in humans, suggesting conservation of functional capability is of greater importance than community composition (Lloyd-Price et al., 2017). While measurements of microbial function are not without limitations, such as higher cost and more complex analysis, use of these metrics to characterize the microbiome are increasing. One such example is predicted metabolic capacity, which utilizes the imputed gene makeup of the bacteria in the sample or metabolite profiles (Langille et al., 2013). In addition, a functioning microbiome produces bacterial metabolites termed “postbiotics” that may directly affect host function (Tsilingiri et al., 2012; Ojeda et al., 2016). Postbiotics are metabolic products of bacteria; in a nutritional context, postbiotics are generated when food that is undigested by the pet is bypassed to the colon to become available to the microbiome. Postbiotics include not only metabolites from bypass carbohydrates, fat and protein, but also microbial derivatives of other compounds, such as secondary plant compounds; these metabolites can have a beneficial or detrimental effect on the host, depending upon the nature of the compound (Table 1). While the gut microbiome clearly has the potential to influence host health, evidence that links specific changes in microbiome structure with changes in host function is still needed (McBurney et al., 2019). Further, validated biomarkers or other surrogate indicators of host function and pathogenic processes based on the microbiome are also needed (McBurney et al., 2019).

**TABLE 1:** Examples of postbiotics that may impact pet health.

**Predominant Type**	**Postbiotic**	**Nutrient substrate (macronutrient class; substrate)**	**Target organ system**	**Mechanism of action**	**References**
Saccharolytic; fermentative	SCFA; acetate	Carbohydrate; indigestible polysaccharides	Neuroendocrine; systemic energy availability	Increase satiety; substrate for hepatic lipogenesis	Macfarlane and Macfarlane, 2012; Canfora et al., 2015; Kasubuchi et al., 2015; Koh et al., 2016
Saccharolytic; fermentative	SCFA; propionate	Carbohydrate; indigestible polysaccharides	Neuroendocrine; systemic energy availability	Increase satiety; substrate for hepatic gluconeogenesis	Macfarlane and Macfarlane, 2012; De Vadder et al., 2014; Canfora et al., 2015; Kasubuchi et al., 2015; Koh et al., 2016
Saccharolytic; fermentative	SCFA; butyrate	Carbohydrate; indigestible polysaccharides	Colon	Energy substrate for colonocytes; epigenetic modulation; colon electrolyte balance, motility, blood flow	Wächtershäuser and Stein, 2000; Leonel and Alvarez-Leite, 2012; Macfarlane and Macfarlane, 2012; Canfora et al., 2015; Bultman, 2017
Proteolytic; putrefactive	Indole, indole-3-propionate	Protein; tryptophan	Colon, neuroendocrine	Bind to the arylhydrocarbon receptor; increase epithelial-cell tight-junction resistance and gut barrier integrity; decrease markers of inflammation; improve host-microbiome immune homeostasis; modulate GLP-1 secretion	Macfarlane and Macfarlane, 2012; Agus et al., 2018; Gilbert et al., 2018; Kim, 2018; Zhao et al., 2018
Proteolytic; putrefactive	Polyamines (spermidine, spermine, putrescine, and cadaverine)	Protein; arginine, lysine	Colon	Delay intestinal epithelial senescence; maintenance of intestinal barrier function through promotion of occludin and cadherin expression	Timmons et al., 2012; Kibe et al., 2014; Michael, 2016
Proteolytic; putrefactive	bSCFA; isobutyrate (2-methylpropionate)	Protein; valine	Colon	Source of energy for colonocytes; refeed starved colonocytes with greater efficiency and rapidity than butyrate	Jaskiewicz et al., 1996; Smith and Macfarlane, 1997; Koh et al., 2016
Proteolytic; putrefactive	Hydrogen sulfide	Protein; cyst(e)ine, methionine, taurine	Colon	Inhibition of mitochondrial metalloproteins; reduction in SCFA butyrate oxidation; associated with ulcerative colitis	Pun et al., 2010; De Preter et al., 2012; Macfarlane and Macfarlane, 2012; Stein and Baley, 2013; Singh and Lin, 2015; Ridlon et al., 2016
Proteolytic; putrefactive	Uremic toxins (Indoxyl sulfate, p-cresyl sulfate, 4-ethylphenyl sulfate, phenylacetylglutamine)	Protein; tryptophan, phenylalanine, tyrosine	Kidney	Proinflammation, exacerbate decline in kidney function	Lau et al., 2018
Lipolytic	10-hydroxy-*cis*-12-octadecenoic acid	Fat; linoleic acid	Oral, Dermis	Increased gingival epithelial barrier integrity; decreased dermal atopy	Kaikiri et al., 2017; Yamada et al., 2018

## Impact of Nutritonal Interventions on the Microbiome of Cats and Dogs

### The Dietary Microbiome of Cats Versus Dogs: Physiologic Differences and Nutritional Evolution

As noted earlier, the compostion of the gut microbiome is generally similar between cats and dogs, with some differences noted. There is a complex relationship between the gut microbiome and the host, which is certainly influenced by the unique anatomy and physiology of cats versus dogs. However, evidence on how the digestive and physiological differences between cats and dogs might affect the microbiome is limited. Nevertheless, intriguing opportunities to study how food influences the health of each species through the production of microbial postbiotics have emerged. For example, when provided with a variety of foods of similar palatability, but different macronutrient content, and allowed to self-select macronutrient intake through free-choice feeding among the foods, the microbial postbiotics generated by cats at their chosen protein level were different from the microbial postbiotics generated by dogs at their chosen protein level (Hall et al., 2018). This resulted in significantly different postbiotics observed between the species; for example, none of the circulating plasma microbial metabolites changed in dogs after the 28-day free-choice feeding period, whereas 16 of 38 metabolite concentrations changed in cats (Hall et al., 2018). These differences between cats and dogs, both in preferred macronutrient intake and in the metabolic response of the microbiome to the selected macronutrient intakes, present unique challenges and benefits for optimizing nutrition intakes for cats and dogs.

One example of the links between the microbiome, nutrition, and digestive physiology is the production of equol, an isoflavone-derived metabolite produced through the microbial metabolism of daidzein. Equol production is known to be enhanced by a high carbohydrate diet (Vázquez et al., 2020). In the free-choice feeding article described previously, circulating daidzein sulfate levels were elevated in cats while equol sulfate levels were not different between cats and dogs (Hall et al., 2018). The authors postulate that the mixed diet chosen by cats (with macronutrient ratios higher in both protein and carbohydrate than those chosen by dogs) would likely have resulted in increased daidzein intakes and an expectation for increased equol production by the cat microbiome. Yet, circulating equol levels were similar in cats and dogs, a finding the authors interpreted as evidence that the microbiomes of cats do not have a similar capacity for equol production as those of dogs (Hall et al., 2018). The links between the microbiome, nutrition, and digestive physiology were seen in this study not only for anti-inflammatory postbiotics such as equol, but pro-inflammatory compounds such as 4-ethylphenyl sulfate, which was higher in dogs compared to cats at baseline, and p-cresol sulfate, which was higher in cats compared to dogs at baseline (Hall et al., 2018).

### Nutritional Interventions and the Microbiome

The degree of digestibility of a food determines the extent to which different nutrients are digested and absorbed by the host. Those nutrients that escape digestion and absorption by the host arrive to the colon and are thus available for microbial metabolism. High digestibility of protein and fat is important when provisioning food for cats and dogs, ensuring that the pet is appropriately nourished. In contrast, the “bypass” food components that are incompletely digested in the upper GI tract serve as nutrient sources for the GI microbiome, playing a significant role in defining its composition and functions. Together, the host and resident GI microbes harbor complementary digestive enzymes by which ingested food is broken down through saccharolysis, proteolysis and lipolysis, nourishing not only the pet but also the microbiome (Figure 1). Changes to the microbiome have been shown to occur quickly in response to dietary interventions (David et al., 2014; Mori et al., 2019). For example, variations in the amount of carbohydrate, protein, and fat that are fed to cats and dogs can have a significant impact on their intestinal microbiota (Mori et al., 2019). This was demonstrated in a recent study evaluating the effect of four commercially available prescription diet regimens on the fecal microbiome in six healthy dogs (Mori et al., 2019). Each dog was fed one of the following four diets for 21 days each: (1) weight-loss diet (high protein, low fat, low carbohydrate, high fiber); (2) low-fat diet (low fat, medium protein, high carbohydrate, low fiber); (3) renal diet (low protein, high fat, high carbohydrate, low fiber); and (4) an allergenic diet (hydrolyzed medium protein, high fat, medium carbohydrate, low fiber). This study revealed several significant differences in the microbiome of dogs fed the weight loss diet versus those fed the allergeneic diet, including significantly decreased proportions of *Actinobacteria* and *Firmicutes* and significantly increased proportions of *Fusobacteria* (Mori et al., 2019). Nevertheless, not every nutritional intervention results in a change to the microbiome composition (Bresciani et al., 2018; Pilla et al., 2019). The following sections summarize evidence for common nutritional interventions with the potential to impact the microbiome of cats and dogs, including probiotics, prebiotics, simple and complex carbohydrates, proteins, and fats.

#### Probiotics

One commonly used approach to improve the gut microbiome, and thus the health of the host, is the use of probiotics. Probiotics have been defined as live microorganisms, which when consumed in adequate amounts as part of a food, may confer a health benefit on the host (Food and Agriculture Organization of the United Nations/World Health Organization, 2002). Many of the probiotics studied in pet health belong to the genera of *Lactobacillus*, *Bifidobacterium* and *Enterococcus* (Weese and Martin, 2011; Schmitz and Suchodolski, 2016; Jugan et al., 2017; Sanders et al., 2019). Probiotics can alter the resident microbiome through several mechanisms, including stimulation of the growth of resident bacteria through metabolic interactions, altering the abundance of pathogenic bacteria, or indirectly through interactions with the host epithelium and epithelial immune system (Derrien and van Hylckama Vlieg, 2015), but successful colonization of the resident microbiome by the probiotic is not universal and depends on characteristics of both the resident microbiome and the host (Derrien and van Hylckama Vlieg, 2015; Zmora et al., 2018) including the host’s dietary regimen (Derrien and van Hylckama Vlieg, 2015). Some have suggested that the bacterial species being utilized in a probiotic should ideally originate from the gut of the host species (Grzeskowiak et al., 2015; Suez and Elinav, 2017); however, this is not true for most probiotics currently marketed for cats and dogs (Grzeskowiak et al., 2015), and no studies comparing the efficacy of dog- or cat-derived probiotics to commercially available strains derived from other species exist. A review of commercially available veterinary probiotics revealed quality issues including inaccurate labels and poor viability (Weese and Martin, 2011); others have raised concerns about safety, though additional research is needed to understand the frequency and severity of adverse outcomes attributed to probiotic use in cats and dogs (Doron and Snydman, 2015).

#### Prebiotics

Historically, the term prebiotic has been defined as “a non-digestible food ingredient that beneficially alters the host by selectively stimulating the growth and/or activity of one or a limited number of bacteria in the colon, and thus improves host health” (Gibson and Roberfroid, 1995). However, it is now understood that any substance that is available to the gut microbiome for fermentation, including bypass nutrients that reach the microbiome such as carbohydrate, protein, amino acids, fat and polpyphenols, can serve as a prebiotic (Gibson et al., 2010). Therefore, prebiotics are now defined as “selectively fermented ingredients that allow specific changes, both in the composition and/or activity in the GI microflora that confer benefits upon host well-being and health” (Gibson et al., 2004, 2010).

#### Complex Carbohydrates

Complex carbohydrates are those that include three or more sugars (i.e., oligosaccharides and polysaccharides). While not all complex carbohydrates provide fiber, those commonly used in the pet food industry include traditional sources of fiber for cats and dogs, such as beet pulp, which contains a mix of insoluble and soluble fiber and cellulose, which is a non-fermentable, insoluble, non-viscous fiber (De Godoy et al., 2013). Other sources of fiber either being utilized or evaluated in pet foods include corn, fruit, rice, oat, and barley fiber; these fibers are sources of resistant starches and soluble fibers that can serve as substrates for the GI microbiome (De Godoy et al., 2013).

**FIGURE 2:**
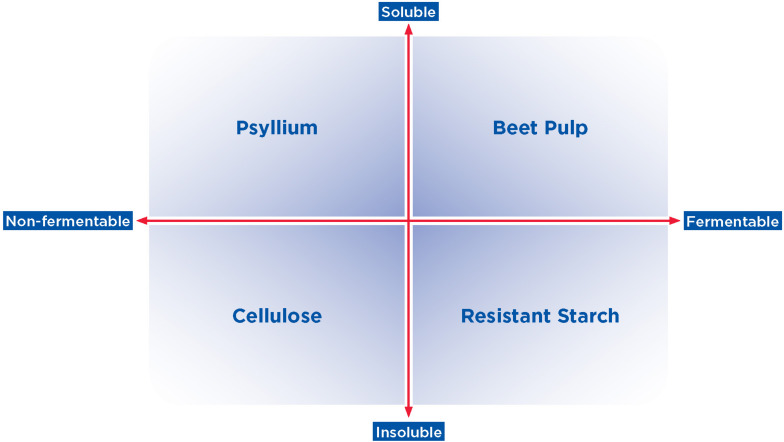
Spectrum of solubility and fermentability of complex carbohydrates and fiber. Common fiber sources used in the pet food industry, such as those shown here, vary in their degree of solubility and fermentability (upper quadrants: more soluble; lower quadrants: less soluble; left quadrants: less fermentable; right quadrants: more fermentable). Examples of fibers that generally represent each combination of solubility and fermentability are shown.

Although multiple classification systems have been suggested for dietary fibers (Institute of Medicine, 2001; Tungland and Meyer, 2002), the characteristics of solubility and fermentability have been proposed as the most appropriate classifications (Tungland and Meyer, 2002; Dhingra et al., 2012). Although many ingredients contain multiple types of fibers, Figure 2 highlights the range of solubility and fermentability that generally characterize common fiber sources used in the pet food industry (Iktor et al., 1989; Naran et al., 2008; Aura et al., 2013; McRorie, 2013; Azad et al., 2014; Chung et al., 2017; Gupta et al., 2018). The physical and chemical properties of the fiber, including particle size and bulk volume, surface area characteristics, and hydration properties determine accessibility of the fiber to microbial degradation, as well as the physiological effects of the fiber (Dhingra et al., 2012) and thus the metabolic fate of the fiber in the digestive tract (Dhingra et al., 2012). The ability of specific saccharolytic microbes to convert fiber to fermentation end products depends both on the chemical linkages present in the dietary fiber and the metabolic capabilities of a given microbe (Rogowski et al., 2015).

The impact of different types of complex carbohydrates on microbiome composition has been evaluated in healthy cats and dogs. For example, studies of resistant starches, or starches not digested or absorbed in the small intestine of a healthy animal (Yang et al., 2017), have been conducted in dogs. These studies have demonstrated that increasing concentrations of potato fiber, which is composed of both resistant and digestible starch, increased the proportion of *Faecalibacterium* in the microbiome (Panasevich et al., 2015). Feeding fructooligosaccharide (FOS) has been shown to increase potentially beneficial *Lactobacillus* and *Bifidobacterium* populations and reduce potentially detrimental *C. perfringens* in one study in dogs (Swanson et al., 2002); a second study suggested FOS interacted with dietary protein resulting in lower measured fecal *Bifidobacterium* when fed with a low protein diet and higher measured fecal *Bifidobacterium* when fed with a high protein diet (Pinna et al., 2018). Other studies in dogs fed diets supplemented with beet fiber showed that those fed higher concentrations of beet pulp had significant changes in gut microbial composition, marked in one study by decreased *Fusobacteria* and increased *Firmicutes* (Middelbos et al., 2010) and in another by increased *Lactobacillus* spp., *Bifidobacterium* spp., *Clostridium coccoides* cluster and *Clostridium leptum* cluster (Kröger et al., 2017), though a third study did not show significant differences (Maria et al., 2017). Finally, a study evaluating a mix of carbohydrate sources (containing 0.07% concentrations of apple pectin, inulin, arabinoxylan-oligosaccharides, resistant starch type III, galactomannan and β-glucan) showed that complex non-digestible carbohydrate substrates and substrate mixtures may have a significant impact on gut microbiota composition and diversity (Chung et al., 2019).

Li et al. (2017) used predicted metabolic capacity of the microbiome to show that carbohydrate-rich foods appeared to favor the growth of bacteria that were enriched in pathways associated with starch digestion and nutrient absorption. In this study, dogs fed a low protein/high carbohydrate diet had an overabundance of pathways associated with carbohydrate digestion/absorption and mineral absorption; this may indicate these pathways are enriched with intestinal bacteria that have the capability of fermenting and utilizing dietary carbohydrates, which can increase mineral bioavailability and promote colonic absorption (Li et al., 2017), In addition, Jackson and Jewell observed the postbiotic products of the microbial population in response to a mixture of soluble and insoluble fibers added to two different background foods (grain rich versus hydrolyzed meat) and noted largely similar postbiotic production regardless of the background food (Jackson and Jewell, 2018). For both foods, fiber inclusion improved stool quality, lowered stool pH, increased beneficial gut microbes, and changed microbial metabolites to suggest improved colonic health. However, the addition of fiber to the hydrolyzed meat food increased SCFA (acetate, propionate, and butyrate) levels typically associated with saccharolysis while the addition of fiber to grain-rich decreased branched SCFAs (bSCFA; 2-methyl propionate, 2-methyl butyrate, and 3-methyl butyrate). This study suggests that the effects of fiber inclusion are dependent upon the host baseline microbial structure and its functional capability.

Numerous studies have examined the impact of other sources of fiber on the microbiome composition of healthy dogs. One recent study evaluated the effects of diets supplemented with a fiber and a prebiotic blend (both with and without saccharin and eugenol) fed to eight adult female dogs for a 14-day period (Nogueira et al., 2019). The addition of the fiber and prebiotic did not affect the richness and diversity of the fecal microbiota compared with a control diet. However, beneficial shifts in fecal fermentative end products that may support gut health were observed, including an increase in fecal SCFA concentrations and a decrease in phenols, indoles, bSCFA (isobutyrate and isovalerate), and ammonia concentrations. Moreover, total dietary fiber digestibility was significantly greater for dogs fed a diet containing the fiber-prebiotic blend with saccharin and eugenol (Nogueira et al., 2019). Similarly, polydextrose, an insoluble fiber with prebiotic properties, has been shown to alter fecal pH and fermentative end products of healthy adult dogs in a beneficial manner, while having little effect on the composition of fecal microbiota (Beloshapka et al., 2012).

Although most prebiotics come from plant fibers, the chitinous exoskeletons of arthropods represent a potential novel source of fiber for dogs (Jarett et al., 2019). In one recent study, 32 male and female beagles from 1 to 8 years of age were fed one of four diets with 0, 8, 16, or 24% of the protein content replaced with whole cricket meal. Similar to the studies above, this analysis determined that each cricket inclusion level had similar effects on the diversity and composition of the gut microbiome of dogs compared with a standard healthy diet without crickets (Jarett et al., 2019). Another novel fiber source, cooked navy bean powder, did not have any significant impact on the microbiome composition of healthy dogs fed a diet containing either 0% or 25% navy beans for 4 weeks (Kerr et al., 2013).

Far fewer studies have evaluated the effects of fiber sources on the microbial composition of healthy cats. One study found that the feline microbiome was primarily unchanged in four cats fed three different diets supplemented by either cellulose, fructooligosaccharides (FOS), or pectin (Barry et al., 2012). Another analysis studied the effects of a diet supplemented with prebiotics on the digestibility of nutrients, fermentative metabolite concentrations, and the GI microbiome of eight healthy adult cats (Kanakupt et al., 2011). These cats were fed diets containing no prebiotic (control), 0.5% short-chain FOS, 0.5% galactooligosaccharides (GOS), or 0.5% short chain FOS plus 0.5% GOS. This study found that short chain FOS, GOS, and short-chain FOS + GOS-supplemented diets significantly increased *Bifidobacterium* spp. concentrations (*P* < 0.05) compared with the control diet (Kanakupt et al., 2011). In addition, cats fed the diet supplemented with short-chain FOS plus GOS had greater fecal concentrations of acetate, butyrate, valerate, SCFAs, and bSCFAs, as well as a lower fecal pH (*P* < 0.05); there was no difference in fecal protein metabolites among the four diets. Overall, this study concluded that small amounts of these prebiotics had beneficial effects on various parameters of digestive health (Kanakupt et al., 2011). Lastly, a study in both healthy cats and dogs found that a commercially available product containing prebiotics (FOS and inulin) did not significantly change the abundance of most fecal bacteria when it was given for 16 days, while also noting that the response to prebiotic administration varied widely among individual cats and dogs (Garcia-Mazcorro et al., 2017).

Subsequent sections in this article will review the impact of a variety of nutrition interventions, including fiber, probiotics, and synbiotics, in various disease states of cats and dogs.

#### Simple Carbohydrates

Although the effects of complex carbohydrates on the microbiome have been well-studied, simple carbohydrates such as monosaccharides and disaccharides can also change the composition of the microbiome. Simple carbohydrates can be found in the diet or generated as the product of polysaccharide digestion in the upper small intestine. In rats, early life exposure to sugar (varying ratios of glucose and fructose) reduced *Prevotella* and *Lachnospiraceae incertae sedis* but increased *Bacteroides*, *Alistipes, Lactobacillus*, *Clostridium sensu stricto*, *Bifidobacteriaceae*, and *Parasutterella* (Noble et al., 2017). Some of these increases, which were independent of monosaccharide ratio, caloric intake, body weight, or adiposity index, have been previously associated with health and/or disease processes, such as metabolic and cognitive disorders. However, the implications of these changes on disorders associated with high sugar consumption require further study in cats and dogs.

#### Protein

Traditionally, protein quality has been defined by the amino acid composition of the protein, as well as digestibility and bioavailability of the protein source. Most pet foods use animal protein sources since they typically contain a more complete complement of amino acids, though plant protein sources are also used. Regardless of the source, dietary protein must undergo proteolysis, or the hydrolysis of intact proteins into individual amino acids, to be absorbed by the animal. In many cases, protein hydrolysis occurs as part of host digestion, though some specialized foods use hydrolyzed protein ingredients to reduce the potential for allergic response and ease digestion. Different protein sources vary not only in their amino acid composition, but also in their micro- and macronutrients, all of which affect the GI microbiome (Madsen et al., 2017). The concepts of protein quality (completeness of the amino acid profile and digestibility), quantity (amount of protein per serving) and quotient (ratio of protein to other energy-containing nutrients such as carbohydrate) are important factors in both host and microbial metabolism, since nutrients not digested and absorbed by the host become available for metabolism by the microbiome in the lower GI tract.

Illustrating the concepts of quality and quotient, there is evidence to suggest that the source of protein (quality) and ratio of protein to carbohydrate (quotient) can influence the composition of the GI microbiome (Jackson and Jewell, 2018). Among healthy dogs, consumption of a food with a hydrolyzed meat protein source and no whole grains resulted in increased diversity and genus-level evenness compared to consumption of a grain-rich food with intact protein, though taxa richness did not differ (Jackson and Jewell, 2018). Furthermore, the addition of fiber to the hydrolyzed meat food resulted in a larger number of significant changes in operational taxonomic units compared to the addition of fiber to the grain-rich food. Because the authors evaluated both intact and hydrolyzed protein sources, the observed microbiome changes cannot be definitively ascribed to the protein source; the ratio of protein to carbohydrate in the foods likely also contributes. Other authors have studied the effect of hydrolyzed protein foods directly. Feeding a hydrolyzed protein food was found to have no impact on the microbiome composition of either healthy dogs or those with food-responsive chronic enteropathy (Pilla et al., 2019). Other groups have evaluated the impact of food that used plant protein sources on the microbiome, and found that feeding an animal protein-free food had no impact on the microbiomes of healthy dogs, while dogs with food responsive enteropathy had increased microbiota richness after consuming the food (Bresciani et al., 2018).

Raw meat-based diets (RMBD) are high protein diets that illustrate the concepts of protein quantity and quotient. Such diets include uncooked ingredients derived from animals (Freeman et al., 2013); these ingredients may include skeletal muscle, fat, internal organs, cartilage, and bones from either farm animals (ruminants, pigs, and poultry), horses, game, or fish, as well as unpasteurized milk and uncooked eggs (Freeman et al., 2013; Fredriksson-Ahomaa et al., 2017) and these diets typically have different macronutrient profiles characterized by higher protein and fat content than commercially produced pet foods. While RMBD diets are generally not recommended by most major veterinary and public health organizations due to the potential for pathogen contamination (American Animal Hospital Association, 2011; American Veterinary Medical Association, 2012; Centers for Disease Control and Prevention, 2017; Food and Drug Administration, 2018), they are increasingly popular. Numerous studies have shown that animals fed RMBDs differ in GI microbiome composition and metabolism compared to those fed extruded, heat-processed foods (Kerr et al., 2012, 2014; Herstad et al., 2017; Kim et al., 2017; Sandri et al., 2017; Algya et al., 2018; Schmidt et al., 2018; Butowski et al., 2019). For example, dogs fed a RMBD exhibited more diverse and abundant microbes in the gut compared with those fed commercial foods; however, as the authors pointed out, dogs fed an RMBD may have a greater risk of opportunistic infection than those fed commercial foods (Kim et al., 2017). Another study reported no significant difference in measures of alpha diversity between dogs fed RMBDs and commercial foods, but found a significant difference in beta diversity between groups; this may have been due in part to the different macronutrient compositions of the two types of foods, with significantly higher protein and fat and lower carbohydrate and fiber content in the RMBDs compared to the commercial foods (Schmidt et al., 2018). Finally, a study compared the microbiome of cats fed an RMBD versus a similar diet with added fiber and a commercially available kibble (Butowski et al., 2019) and found that 31 bacterial taxa were affected by diet. *Prevotella* was abundant in microbiomes of cats fed the kibble diet, *Clostridium* and *Fusobacterium* were abundant in microbiomes of cats fed the RMBD, and *Prevotella* along with a group of unclassified *Peptostreptococcaceae* were abundant in the microbiomes of cats fed the RMB with added fiber. Based on the available evidence to date, research on RMBDs does not show clear benefit on the gut microbiome and may increase the risk of pathogen exposure.

Ingestion of moderate quantities of highly digestible protein balances the concepts of quantity, quality, and quotient, optimizing protein utilization by the pet, while ingestion of large amounts of poorly digestible protein increases presentation of dietary protein to bacterial populations in the colon, where it undergoes putrefaction. Putrefaction is the process of microbial decomposition of amino acids into postbiotic products, some of which have been implicated in the initiation and progression of certain inflammatory diseases, such as atopy (Nylund et al., 2015), chronic renal failure (Niwa et al., 1997), and chronic enteritis (Nikolaus et al., 2017; Table 1). In short, it is both the total amount of protein ingested (quantity), ratio of protein to other energy-containing ingredients such as carbohydrates (quotient), and the digestibility and amino acid composition of protein (quality) in the diet that determine the amount and amino acid composition of the bypass digesta, and the subsequent availability of nitrogenous waste (e.g., ammonia and urea) presented for microbial metabolism.

#### Fat

Much less is known regarding the role that fat quantity plays in influencing the microbiome than protein and carbohydrate. Most of the information available describes the impact of high dietary fat on the microbiota of either humans or mice (Shen et al., 2014; Martinez et al., 2017). These reports show a clear link between high-fat diets (45−60% of daily caloric intake) and a rapid and dramatic shift in the microbiota within 2−3 days of starting consumption of the high-fat diet. Furthermore, decreases in beneficial postbiotic SCFAs and increases in detrimental hydrogen sulfide (H_2_S) are found in obese individuals (Martinez et al., 2017), though in the context of metabolic syndrome, it is difficult to isolate the specific effect of fat intake. Thus, the pro-inflammatory nature of dietary fat may influence microbiome composition, perhaps through host-microbiome immune-mediated homeostasis. Substantial additional research is needed to better understand the mechanisms underlying these effects.

Fatty acids are underappreciated prebiotics that have the potential to influence health and physiology through their effects on the GI microbiome. Emerging research has indicated a role for gut microbial metabolism of linoleic acid to produce postbiotic metabolites with health-promoting effects. For example, a bacterial metabolite of linoleic acid has been shown to prevent the impairment of the epithelial barrier that is caused by periodontopathic bacteria (Yamada et al., 2018). Future research on the targeted delivery of lipids to the colon will determine if this is a successful strategy to influence the production of fatty acid postbiotics with the potential to improve animal health.

## Current Evidence: the Role of Nutrition in Influencing Cat and Dog Health Through Changes to the Gi Microbiome

The GI microbiome influences an array of physiological and immunological functions either directly or indirectly, including energy homeostasis and metabolism, endocrine signaling, inhibition of enteropathogenic colonization, and immune function regulation (Nicholson et al., 2012; Wilson and Nicholson, 2015). Consequently, disruptions to the composition of the GI microbiome can lead to detrimental health consequences, including inflammatory enteropathies (e.g., inflammatory bowel disease), allergy, constipation, oral disease (i.e., periodontal disease), obesity, diabetes, and kidney disease (Nicholson et al., 2012). Nutrition has the potential to both affect the disease condition directly through provision of substances like macro- and micronutrients, as well as indirectly by changing the microbiome, while the microbiome in turn influences the response to nutrition (Suez and Elinav, 2017). Here, we consider current evidence for the role of nutrition in influencing cat and dog health through changes to the GI microbiome.

### Inflammatory Enteropathies

Because the GI microbiome influences the environmental habitat of the GI tract and vice versa, the incidence and progression of microbiome-associated chronic GI enteropathies, particularly inflammatory bowel disease (IBD), has been the subject of considerable research. Most research reported to date involving the microbiome in cats and dogs with GI enteropathies involves dogs, with relatively fewer studies in cats. Dogs with chronic enteropathy (CE) have been shown to have lower concentrations and altered patterns of SCFAs, as well as changes in the fecal microbiota compared to healthy dogs (Minamoto et al., 2019). With respect to cats, it has been shown that the GI microbiota in those with IBD is altered compared to healthy cats (Garraway et al., 2018). One study also found that *Fusobacterium* spp. are elevated in the ileum and colon of biopsies from cats with small cell GI lymphoma compared to those with IBD; however, it’s not yet clear whether this change plays any role in the development of GI lymphoma (Garraway et al., 2018). While a number of previous reviews have discussed this topic at length (Heilmann and Allenspach, 2017; Redfern et al., 2017; Roth-Walter et al., 2017; Yogeshpriya et al., 2017; Barko et al., 2018), few authors have explicitly evaluated the impact of macronutrient nutrition (nutrition supplied by carbohydrates, protein, and fat), digestion, and food processing on the pet microbiome and its subsequent effect on health outcomes in IBD. Dysbiosis, which is more common in animals with IBD, compared to those without the disease, is characterized by decreased microbiome diversity in general, and a reduction in species that produce SCFAs in particular. One study examined ileal and colonic mucosal microbiota samples from dogs with IBD and found that they manifested increased *Enterobacteriaceae* and *E. coli* bacteria attached to epithelia or invading the intestinal mucosa at these sampling sites (Cassmann et al., 2016). These findings are consistent with that of another recent study which also found increased *E. coli* in the colonic mucosa of dogs with CE, as well as decreased *Helicobacter* spp. and *Akkermansia* spp. (Giaretta et al., 2020). Interestingly, a recent review of the literature noted that compared to humans, *Akkermansia* spp. are not abundant in the GI tracts of cats and dogs, suggesting that these bacteria do not have a significant role in the microbial degradation of mucus in these animals (Garcia-Mazcorro et al., 2020).

Two endoscopic studies examined the impact of dietary intervention in dogs diagnosed with IBD (Marchesi, 2017; Kalenyak et al., 2018). These studies found that an intervention with an elimination diet (i.e., a diet that eliminates foods/ingredients suspected of causing adverse effects) altered the gastrointestinal microbiomes that reside in the mucosal layer of the duodenum and colon (Kalenyak et al., 2018), and changes in food alone were efficacious in reducing signs of IBD in almost all dogs with mild disease (Marchesi, 2017). Both novel proteins and hydrolyzed proteins were effective (Marchesi, 2017; Kalenyak et al., 2018), but hydrolyzed proteins were slightly more effective in reducing the signs of IBD (Marchesi, 2017). Further, the Effect of Nutritional Therapy on Microbiome in Canine Enteropathy (ENTiCE) study also analyzed the effects of a hydrolyzed protein diet (Wang et al., 2019) on the microbiome and health of 29 dogs with CE. Dogs enrolled in the study were switched from their current diet to a commercially available therapeutic hydrolyzed protein diet. Following 2 weeks on this therapeutic diet, 69% of dogs experienced a rapid remission of their CE, which was sustained for the entire 6-week duration of the study. In addition, remission of CE was associated with an improvement in the structure of microbiota and increased levels of secondary bile acids (Wang et al., 2019).

Another study of dogs with IBD fed an elimination diet showed that the microbiota in dogs whose IBD signs and symptoms resolved differed from those that did not experience improvement. Those dogs who responded to the diet had a predominance of *Bilophila* and *Burkholderia* and enrichment in *Bacteroides*, while those that did not improve had a greater abundance of *Neisseriaceae* (Kalenyak et al., 2018). *Burkholderiales* has not been shown to be associated with chronic enteropathies in dogs and *Bacteroides* can be either protective or virulent (Kalenyak et al., 2018). A study conducted in dogs with food-responsive CE found a small increase in bacterial richness as indicated by species diversity (alpha diversity) in response to feeding a hydrolyzed protein food paired with a synbiotic, though no change in microbial composition (beta diversity) was seen (Pilla et al., 2019).

Several studies have evaluated the impact of probiotic treatment in dogs diagnosed with IBD. Results from these analyses suggest that multi-strain probiotic treatments facilitated clinical remission in dogs diagnosed with IBD and were associated with several other benefits, including anti-inflammatory and anti-proliferative effects, increased tight junction protein expression, and up-regulation of polyamine levels (Rossi et al., 2014, 2018; White et al., 2017). With respect to the composition of the microbiome, dogs with IBD treated with probiotics have exhibited significant increases in *Faecalibacterium* spp. and *Lactobacillus* spp. versus those receiving standard treatments, however, these results were not consistent between studies (Rossi et al., 2014; White et al., 2017). In addition to beneficial effects in IBD, several double-blind, placbo-controlled studies have demonsrated that probiotic administration can also reduce diarrhea in cats and dogs (Bybee et al., 2011; Gómez-Gallego et al., 2016).

Additional well-designed and controlled trials are needed to assess the interrelationships among nutrition and the microbiome in the management of IBD in cats and dogs.

### Allergy

Food allergies manifesting with GI symptoms in animals are often mistaken for IBD and vice-versa due to the overlap of symptoms, most noticeably diarrhea. Although few studies have been conducted in pets and humans, it has been found that in adult humans, peanut and tree nut allergies are associated with higher *Bacteroides* and reduced *Clostridiales, Prevotella*, and *Ruminococcaceae* (Hua et al., 2016). While nut allergies may be less relevant for cats and dogs, this report suggests that microbiome alterations may be associated with allergic diseases.

Since allergies are essentially immune disorders and the GI microbiome has been shown to have both pro- and anti-inflammatory properties depending on the colonizing organisms, targeting the GI microbiome may represent a logical approach for treating systemic allergies (Pascal et al., 2018) and this has been the subject of several animal studies (Thorburn et al., 2015; Aoki-Yoshida et al., 2016; Kim et al., 2016; Kumar et al., 2017). In dogs, the addition of probiotics was found to result in increased fecal concentrations of acetate and butyrate and a reduction in ammonia, as well as improved cell-mediated immune responses to an antigenic challenge (Kumar et al., 2017; Pilla et al., 2019). However, the immune modulating effects of probiotics have not been demonstrated consistently across studies in dogs with food-responsive CE or diarrhea (Sauter et al., 2006; Schmitz et al., 2015).

A validated model of canine atopic dermatitis found that early exposure to probiotics (*Lactobacillus rhamnosus*) significantly decreased allergen-specific IgE and partially prevented atopic dermatitis in the first 6 months of life (Marsella, 2009). A follow-up of this study showed that these effects persisted for 3 years after discontinuation of the probiotic (Marsella et al., 2012). A randomized control trial of dogs with confirmed atopic dermatitis reported that probiotic (*Lactobacillus sakei*) administration for 2 months significantly reduced atopic disease severity in all dogs who received it (Kim et al., 2015a). It should be noted that the vast majority of research evaluating the use of probiotics in treating systemic allergies has been conducted in humans (Osborn and Sinn, 2007a, b; Bresciani et al., 2018; Pilla et al., 2019).

### Constipation

The prevalence of constipation among cats and dogs is not well-described, and defecation frequency is difficult for pet owners to quantitate (Davenport et al., 2010). Nevertheless, constipation may be a relatively common clinical problem in pets, particularly in cats (Chandler, 2013).

No clear link between constipation and GI microbiome composition, including the abundance of specific bacterial groups, has yet been identified (Mancabelli et al., 2017; Rossi et al., 2017), and the precise mechanisms by which intestinal microbiota may impact GI sensory and motor functions are unclear (Zhao and Yu, 2016). Nevertheless, evidence suggests a link, albeit mainly from human studies (Tigchelaar et al., 2016; Vandeputte et al., 2016). Studies in animals are more limited. In one study of 20 cats, no significant differences were observed in the limited number of bacterial taxa analyzed by PCR between cats with and without constipation (Rossi et al., 2017).

The mechanistic relationship between the GI microbiome and constipation is even less studied and the impact of specific bacterial taxa on GI motility are far from conclusive. One *in vitro* study has suggested that decreased abundance of *Clostridium*, *Lactobacillus*, *Desulfovibrio*, and *Methyobacterium* may influence GI motility through an influence on the serotonin receptor, contributing to development of chronic constipation (Cao et al., 2017). The production of SCFAs, such as acetate and butyrate, by the gut bacteria has also been suggested as a means of altering serotonin availability, which thereby affects motility and secretion in the gut (Spohn and Mawe, 2017). In addition, certain GI bacterial strains have been shown to produce serotonin from tryptophan (O’Mahony et al., 2015). This opens a new avenue of treatment for not only constipation, but for other conditions affected by serotonin levels. However, it also highlights the potential for unintended adverse events from therapies for unrelated conditions, such as serotonin reuptake inhibitors for psychiatric disorders, which may inadvertently affect GI serotonin levels.

Available dietary therapies for constipation have been reviewed (Zhao and Yu, 2016), and mainly focus on fiber and probiotics. Fiber has been shown to modulate the intestinal microbiota of cats (Pinna et al., 2014) though in the limited studies of fiber interventions for feline constipation, microbiome analyses were not performed (Freiche et al., 2011). An intervention trial in cats with constipation evaluated a probiotic (SLAB51) containing multiple strains, including: *Streptococcus thermophilus* DSM32245; *Lactobacillus acidophilus* DSM32241; *Lactobacillus plantarum* DSM32244; *Lactobacillus casei* DSM32243; *Lactobacillus helveticus* DSM322422; *Lactobacillus brevis* DSM27961; *Bifidobacterium lactis* DSM32246 and *B. lactis* DSM32247 (Rossi et al., 2017). Significant decreases in a feline CE activity index were observed, along with improvements in fecal scores and mucosal histology. After treatment, there was a significant increase in populations of *Streptococcus* and *Lactobacillus* and a trend for an increase in *Bifidobacterium* and *Bacteroidetes* (Rossi et al., 2017). The functional impact of the above changes in the bacterial taxa of these cats was not determined.

Although there are limited data in cats and dogs, several reviews have summarized studies on the beneficial impact of probiotics on constipation in humans (Chmielewska and Szajewska, 2010; Miller and Ouwehand, 2013; Dimidi et al., 2014; Ford et al., 2014; Korterink et al., 2014). In addition, a more recent study has evaluated the mechanism of action for probiotics on gut motility (Dimidi et al., 2017); a second study evaluated the effect of probiotics on spontaneous bowel movements in constipated patients (Kim et al., 2015b). Overall, these studies support the use of probiotics for constipation, reducing GI transit time, increasing stool frequency, improving stool consistency, and ameliorating GI symptoms (Miller and Ouwehand, 2013; Dimidi et al., 2014; Ford et al., 2014; Kim et al., 2015b). However, not all studies demonstrated probiotics to be effective in the treatment of constipation (Chmielewska and Szajewska, 2010; Korterink et al., 2014). A systematic review concluded that there was insufficient evidence supporting the use of probiotics in functional constipation (also known as chronic idiopathic constipation) (Chmielewska and Szajewska, 2010) and a meta-analysis found that while probiotics may improve abdominal pain in children with functional GI disorders, they have not been proven effective in childhood constipation (Korterink et al., 2014). Potential mechanisms for these effects center on the interactions between the gut luminal environment, immune system, enteric nervous system, and central nervous system, all of which are highly interrelated and influence gut motility (Dimidi et al., 2017). Additional research evaluating the impact of nutrition inteventions on the microbiome in cats and dogs with constipation is warranted.

### Oral Health

Reported to affect 76% of dogs and 68% of cats, dental disease is the most common disease in pets (Banfield Animal Hospital, 2016). Research indicates 6 bacterial phyla consistently dominate the oral microbiome of clinically healthy dogs and cats: *Actinobacteria, Bacteroidetes, Firmicutes, Fusobacteria, Proteobacteria*, and *Spirochetes* (Davis, 2016). The oral microbiome in cats and dogs with and without documented periodontal disease has been evaluated (Isaiah et al., 2017; Whyte et al., 2017). Overall, these studies suggest that dental disease is associated with a replacement of health-associated taxa with more pathogenic strains.

For example, in cats without periodontal disease, studies have shown that the most common phyla sampled have consistently been *Bacteroidetes, Firmicutes*, and *Proteobacteria* (Sturgeon et al., 2014; Dewhirst et al., 2015; Harris et al., 2015; Adler et al., 2016; Older et al., 2019), while in those with periodontitis and/or gingivitis, a high degree of variation in pathogenic bacteria has been reported, with little similarity across studies (Perez-Salcedo et al., 2013; Harris et al., 2015; Whyte et al., 2017).

On the other hand, in dogs without periodontal disease, the *Bacteroidetes, Firmicutes*, and *Proteobacteria* phyla account for the major phyla among the studies reviewed (Dewhirst et al., 2012; Davis et al., 2013; Sturgeon et al., 2013; Holcombe et al., 2014; Oh et al., 2015; McDonald et al., 2016; Isaiah et al., 2017), although *Actinobacteria* was also reported to be predominant in some studies (Davis et al., 2013; Oh et al., 2015). Studies in dogs with periodontal disease have found varying microbiome composition, depending on the specific stage of oral disease. In one study of dogs with mild periodontitis and/or gingivitis, *Lachnospiraceae*, *Clostridiales*, *Peptostreptococcaceae*, *Peptococcus*, and *Corynebacterium canis* spp. were highly prevalent (Davis et al., 2013). Another study identified *Streptococcus sanguis*, *Peptostreptococcus* spp., *Escherichia coli*, *Proteus mirabilit*, *Veilionella* spp., *Staphylococcus aureus*, *Streptococcus salivarius*, *Actinomyces* spp., and *Actinomyces viscosus* in dogs with advanced stages of periodontopathy (Polkowska et al., 2014). Periodontal disease has been shown to be associated with systemic disease in dogs (Pereira Dos Santos et al., 2019).

A primary distinction among commercial diets of cats and dogs is whether the food is dry or wet. Dry diets are often fed to promote oral health because of the abrasive nature of the dried kibble. Although such foods have been shown to reduce plaque and gingivitis, their impact on the microbiome was not evaluated (Logan et al., 2002). A study of community cats fed exclusively a diet of dry (highly refined, cereal-based dehydrated rations) or wet (canned, sachet, and/or fresh meat combinations) food demonstrated differences in the oral microbiome over time, but could not determine if these differences affected the risk of periodontal disease because the study was not powered to evaluate this endpoint (Adler et al., 2016). However, cats fed the dry diet had a more diverse oral microbiome, with enrichment of bacteria associated with both oral health and periodontal disease (higher abundances of *Porphyromonas* spp. and *Treponema* spp.).

### Obesity and Weight Management

Obesity in pets is a significant problem. According to a recent report by the Association for Pet Obesity Prevention, 60% of cats and 56% of dogs in the United States are classified as overweight or obese (Association for Pet Obesity Prevention, 2019). Obesity is also associated with a variety of other conditions, such as diabetes mellitus (DM), osteoarthritis, cardiovascular disease, skin disorders, and decreased lifespan (German, 2006; Tarkosova et al., 2016; Phillips et al., 2017). Several factors can predispose an animal to obesity, including the GI microbiome, genetics, neutering, decreased activity levels, and high fat and high energy diets (Zeng et al., 2014; Hamper et al., 2016; Davison et al., 2017).

Several reports have found that the composition of the GI microbiome differs between obese and lean cats and dogs (Kieler et al., 2016; Forster et al., 2018). Specifically, bacteria belonging to the phylum *Actinobacteria* and the genus *Roseburia* were significantly more abundant in obese dogs compared with lean dogs (Handl et al., 2013; Forster et al., 2018). In cats, *Clostridium* cluster XIVa groups, *Bacteroidetes*, and *Fusobacteria* were less abundant in obese and overweight cats compared with lean cats, but the abundance of bacteria belonging to *Enterobacteriaceae* and *Clostridium* cluster IV groups were higher in obese and overweight cats (Kieler et al., 2016). Moreover, multiple studies have found that different diets (e.g., high protein, protein/carbohydrate ratios) have different effects on the GI microbiome in obese versus lean animals (Li et al., 2017; Xu et al., 2017; Coelho et al., 2018). For example, one study showed that the microbiome of overweight dogs was more sensitive to dietary intervention, compared with lean dogs (Coelho et al., 2018). In response to being fed a high-protein/low carbohydrate diet for 4 weeks, overweight dogs experienced a significantly greater shift in microbial composition from baseline compared with lean/normal dogs, and that this was driven by greater variation in the abundance of *Lactobacillus*, *Prevotella*, *Streptococcus*, and *Turicibacter* (Coelho et al., 2018).

The GI microbiome has been implicated in the development of obesity through its direct effects on the gut and its indirect influences on distal organs (Leong et al., 2017). The gut microbiota have been shown to influence the metabolism of bile acids; free bile acids resulting from bacterial metabolism can inhibit the growth of bacterial populations, such as *Lactobacilli* and *Bifidobacteria*, which are thought to be protective against obesity (Kurdi et al., 2006). In addition, several bacteria (e.g., *Clostridium scindens*, *Clostridium hylemonae*, and *Clostridium hiranonis*) in the gut contain the genes implicated in bile acid metabolism (Doden et al., 2018). Since bile acids are believed to contribute to gut hormone secretion and glucose and lipid metabolism, the effects of the GI microbiome on bile acids may also influence these processes (Leong et al., 2017). The gut mucosal barrier is affected by the GI microbiome, which can lead to increased inflammation, a known contributor to weight gain (Leong et al., 2017). Disruptions in the GI microbiome have been shown to inhibit lipoprotein lipase, which leads to excess deposition of triglycerides in adipose tissue and the liver, pancreas, and heart (Leong et al., 2017).

A “one-health perspective” has been proposed to address the problem of obesity in both humans and pets because of interdependencies among diet, physical activity, genetics, metabolism, and the GI microbiome related to weight gain (Chandler et al., 2017). Numerous dietary modifications have been proposed to reduce weight and are well-documented in the literature, including the elimination of high-energy density foods, altering the macronutrient composition of the diet (i.e., high-protein, high fat, and/or high fiber diets), and adding dietary diacylglycerols and probiotics (Astrup et al., 2015; Kathrani et al., 2016; Alexander et al., 2017; Vadiveloo et al., 2017). However, current weight management guidelines for cats and dogs do not take into consideration the GI microbiome (Brooks et al., 2014). In fact, it has been found that about half of obese cats and dogs will regain weight after a weight-loss program unless they continue to be fed purpose-formulated weight management diets rather than maintenance diets (German et al., 2012; Deagle et al., 2014), suggesting that continued feeding of specifically formulated diets may be required for weight loss maintenance.

A few studies have investigated microbiome changes associated with weight loss (Kieler et al., 2017; Salas-Mani et al., 2018). One study analyzed 18 obese dogs fed a restrictive commercial high-protein/high-fiber diet. Eight of the 18 dogs were also enrolled in an exercise program (Kieler et al., 2017). Comparable weight loss was experienced in both groups, with no differences in the microbiome between those with added exercise and those managed by diet alone. In both groups, *Megamonas* abundance was found to negatively correlate with weight loss, while lower *Ruminococcaceae* populations was associated with faster (≥1% per week) weight loss. In addition, acetic and propionic acid concentrations decreased in those dogs with faster weight loss (Kieler et al., 2017). Because *Megamonas* and *Ruminococcaceae* produce these acids, these results suggest that a GI microbiome that produces these SCFAs may negatively affect weight loss in dogs (Kieler et al., 2017). A similar small study demonstrated that a restricted low-fat/high-fiber diet for 17 weeks significantly increased GI microbiome diversity in 6 obese beagles (Salas-Mani et al., 2018). At the end of this study, beagles fed this diet also had increases in *Allobaculum* and decreases in *Clostridium*, *Lactobacillus*, and *Dorea*. However, one study evaluating the impact of a moderate-protein/high-fiber diet on body weight and fecal microbiota of obese cats (Pallotto et al., 2018) yielded findings that are inconsistent with the above results. Eight male adult domestic cats were fed a restricted diet tailored to achieve a weight loss of approximately 1.5% of body weight per week for 18 weeks. At week 18, mean body weight decreased by nearly 20% compared with baseline. Although weight loss was associated with a greater proportion of *Actinobacteria* and lower proportion of *Bacteroidetes*, microbiome diversity was not significantly different (Pallotto et al., 2018). Moreover, a similar study found that a standard weight loss diet for 10 weeks had only a small impact on bacterial fecal microbiota in 14 obese cats, compared with 17 lean cats (Tal et al., 2019). Thus, more studies are needed to better understand the relationship between weight loss and fecal microbiota in cats.

The mechanism by which the GI microbiome affects changes in weight is not known. However indole has been reported to modulate (first increase and then over time decrease) the secretion of glucagon-like peptide-1, which stimulates insulin secretion (Chimerel et al., 2014; Table 1) and aids weight loss by delaying gastric emptying, inducing satiety, and decreasing food intake (Woods et al., 2004). Similarly, it was found that proteins secreted by *E. coli* directly activate host satiety pathways, stimulating GLP-1 release, and when these proteins were intraperitoneally administered to rats, they exhibited decreased food intake compared with controls (Breton et al., 2016).

Prebiotics have also been shown to be a potential dietary intervention for promoting health in overweight or obese cats and dogs (Alexander et al., 2018). Although many studies have evaluated fiber intake in relation to weight loss, only a subset included evaluations of the microbiome. One recent study examined the effects of the prebiotic inulin-type fructans on fecal microbiota, metabolites, and bile acids in overweight dogs (Alexander et al., 2018). In this study, nine overweight beagles were fed the same diet twice daily and received treatment over three 14-day periods, separated by a 14-day washout, with one of the following: (1) non-prebiotic control (cellulose); (2) low-dose prebiotic (0.5% of diet); (3) high-dose prebiotic (1% of diet). Each dog received all three treatments over the course of the study. Incremental area under the curve (IAUC) for glucose and insulin was numerically lower in dogs fed the high-dose prebiotic, suggesting a beneficial effect. However, IAUC for glucose, insulin, and GLP-1 was not statistically different among the 3 treatments. Although prebiotic treatment had minimal impact on fecal microbiota and metabolites, some beneficial shifts occurred, including greater concentrations of fecal SCFAs. In addition, prebiotic treatment with inulin resulted in an increase in relative abundance of some members of the phylum *Firmicutes* and a decrease in the relative abundance of some *Proteobacteria* (Alexander et al., 2018). Additional studies in cats and dogs are needed to determine the mechanisms for the shifts in the GI microbiome and bile acid (BA) pool observed with prebiotics.

### Diabetes

Diabetes mellitus (DM) is increasing in prevalence in cats and dogs (Banfield Animal Hospital, 2016). Between 2006 and 2015, the prevalence of DM increased by 79.7% in dogs (from 13.1 to 23.6 per 10,000) and by 18.1% in cats (from 57.2 to 67.6 per 10,000) (Banfield Animal Hospital, 2016). While dogs almost exclusively have type 1 DM, cats are more likely to have type 2 DM. Advances in microbiome research in various model systems indicate that the GI microbiome plays a role in extra-intestinal diseases such as diabetes and obesity (Blake and Suchodolski, 2016). Altered gut microbiota composition has been associated with the development of type 2 DM in cats and dogs (Jergens et al., 2019; Kieler et al., 2019). For example, one recent study showed that dogs with naturally occurring type 1 DM have intestinal dysbiosis and altered concentrations of fecal unconjugated BAs, exhibiting similar patterns to that in humans with type 2 DM (Jergens et al., 2019). In addition, cats with DM have also been shown to have a significant decrease in gut microbial diversity and a loss of butyrate-producing bacteria, compared with healthy cats of the same age (Kieler et al., 2019).

In humans, it has been shown that a lack of appropriate microbiome-dependent maturation of the immune system may exacerbate a genetic predisposition to type 1 DM (Knip and Honkanen, 2017). If an appropriately mutualistic host-microbiome immune complement system does not develop during the critical neonatal “window of opportunity,” the resultant dysbiotic microbiome predisposes genetically susceptible individuals to clinically progressing type 1 DM (Knip and Honkanen, 2017). The importance of microbiome-selective nourishment (e.g., milk oligosaccharides) to facilitate the development of immune competency is a growing field of investigation (Walker and Iyengar, 2015). A number of autoimmune diseases may be related to the inadequate co-development of the microbiome and the host’s immune function, especially with regard to T-regulatory cells and a balanced TH1/TH2 immune response. Early-life diet may play an important role in delivering prebiotic substrates capable of facilitating host-microbiome mutualism.

Not surprisingly, drugs used to treat diabetes have been shown to affect the GI microbiome. In one study, the antidiabetic agent metformin modified the GI microbiome by significantly enhancing the abundance of phylum *Bacteroidetes* (Lee and Ko, 2014). The efficacy of other drugs used to treat diabetes, such as acarbose and metformin, have also been shown to be mediated through the microbiome (Forslund et al., 2015; Gu et al., 2017). Microbiome-based approaches may need to be personalized to effectively manage metabolic-related diseases such as DM to account for inter-individual variation in microbiome composition and function (Shapiro et al., 2017).

The evidence supporting the use of probiotics in DM suggests that probiotics may act to reduce inflammatory responses and oxidative stress and increase the expression of adhesion proteins within the GI epithelium to reduce intestinal permeability (Gomes et al., 2014). These mechanisms have been suggested to result in increased insulin sensitivity and reduced autoimmune responses. One study of 256 women found that probiotic interventions early in pregnancy reduced the rate of gestational diabetes (Barrett et al., 2014). A probiotic containing *Bifidobacterium* was also shown to improve glucose tolerance in a diabetic mouse model (Stenman et al., 2014). Although not feasible for cats and dogs, patients with type 2 DM and/or hypertension who followed a strictly vegetarian diet based on plant sources with high carbohydrates and fiber had reductions in hemoglobin A1c and improvements in fasting and postprandial glucose levels. These effects were also accompanied by a reduction in the *Bacteroidetes/Firmicutes*-ratio in the GI microbiome (Kim et al., 2013).

Because macronutrient profile affects the composition of the GI microbiome, and the associated functional products of the microbiome impact host health, it is logical to approach the management of DM in cats and dogs through nutrition. Although data are currently limited, several studies are underway to further investigate the effects of prebiotics on metabolic diseases. Taken together, there is a clear need to better understand how different nutrition interventions can impact GI microbiome composition and function in DM, which will inform the development of more effective nutritional interventions for DM.

### Kidney Disease

Chronic kidney disease (CKD) is one of the most common diseases in cats and dogs (Polzin, 2007), yet little research has been published to date on the GI microbiome and kidney disease in these species. However, one recent study showed that cats with CKD had decreased richness and diversity of the fecal microbiome compared with healthy cats (Summers et al., 2019), which is consistent with prior studies of the intestinal microbiome in humans with CKD (Hida et al., 1996; Vaziri et al., 2013).

Research to determine the cause and effect relationship between dysbiosis and kidney disease is ongoing. The underlying microbial composition is believed to affect the susceptibility of individuals to CKD, with evidence supporting this link found in cats and dogs. For example, an increased risk of kidney disease has been reported in animals with severe periodontal disease (Glickman et al., 2011; Finch et al., 2016). Factors such as slow GI transit time (Wu et al., 2004), impaired protein assimilation (Bammens et al., 2003), and decreased consumption of dietary fiber (Krishnamurthy et al., 2012), also have been associated with dysbiosis in patients with kidney disease.

Nephrolithiasis, or kidney stones, is a condition common in cats and dogs that shortens the lifespan of cats by about 3 years on average (Hall et al., 2017). The majority of kidney and bladder stones in animals are composed of calcium oxalate or magnesium ammonium phosphate (struvite), and development of stones is a complex process, involving genetic and environmental factors (Mehta et al., 2016). Results of studies in animals and humans evaluating the use of probiotics for reducing kidney stones have been mixed (Mehta et al., 2016; Lieske, 2017; Sadaf et al., 2017). Studies in both humans and animals have shown that *Oxalobacter* and *Lactobacillus* spp. prevent stone formation through the degradation of oxalate salts (Sadaf et al., 2017). More research on the impact of the GI microbiome on kidney function and kidney disease in cats and dogs and the impact of nutrition on these effects is needed.

## Emerging Evidence: Role of Microbiome Function and Postbiotic Effects on Cat and Dog Health

The majority of the pre-existing literature described earlier in this article has focused on associations between compositional changes in the GI microbiome and adverse health effects, including the presence of disease. However, accumulating research suggests that future nutrition interventions that produce changes in microbiome function, which is evident from postbiotic metabolites, may provide significant beneficial impacts on health. Function-based approaches lay the foundation for microbiome-based therapeutics (Suez and Elinav, 2017), a concept based on the fact that many interactions between the host and microbiome are mediated by a wide variety of metabolites secreted, degraded or modified by the microbiome, including fatty acids, amino acids, bile acids, vitamins and polysaccharides (Suez and Elinav, 2017). These metabolites create a signaling network that impacts the host, the microbiome and their inter-dependent functions; therapies based on these metabolites offer the advantage that they target a point downstream of the microorganisms which circumvents inherent compositional differences, as well as the challenges of interventions aimed at creating compositional changes, such as colonization resistance to probiotics (Suez and Elinav, 2017). Nutrition interventions involving microbiome-based metabolites may benefit the host by restoring host metabolic or signaling pathways that have been altered in diseases linked to the microbiome, or they may support a shift in the microbiome composition in a way that reduces the likelihood of disease development (Suez and Elinav, 2017). The subsequent paragraphs provide an overview of emerging evidence for the role of nutrition to influence the health of cats and dogs through changes to microbiome function and postbiotics (see Table 1).

### Protein

Although substantial evidence indicates that reducing excessive putrefaction of bypass protein may provide benefit, there appears to be a role for some putrefactive metabolites to contribute to improved host physiological function. Intriguingly, some putrefactive products may provide health benefits (Table 1). For example, indole, a degradation product of the amino acid tryptophan, has been shown to improve host-microbiome immune homeostasis (Alexeev et al., 2018), decrease markers of inflammation, and increase epithelial-cell tight-junction resistance (Bansal et al., 2010), while the related metabolite indole-3-propionate improves gut barrier integrity (Venkatesh et al., 2014). Conversely, indole has been shown to be a potent co-carcinogen in rats (Dunning and Curtis, 1958; Sims and Renwick, 1983) and hamsters (Oyasu et al., 1972) and is metabolized to 3-indoxyl sulfate, 5-hydroxyindole sulfate and 7-hydroxyindole sulfate, which stimulate the progression of glomerular necrosis and renal failure in uremic patients (Niwa et al., 1997; Barreto et al., 2009; Lisowska-Myjak, 2014). As in humans, indoxyl sulfate is an important uremic toxin in cats and dogs that increases with the severity of renal disease (Wong et al., 2014; Barrios et al., 2015; Cheng et al., 2015). Enhancement of polyamine production has been proposed to abrogate the detrimental impact of aging on intestinal epithelia (Kibe et al., 2014). Finally, bSCFAs derived from putrefaction of branched chain amino acids can refeed starved colonocytes with an efficiency not observed with straight SCFAs (Jaskiewicz et al., 1996). Therefore, bypass protein influences the balance of saccharolytic fermentation versus putrefaction in the gut microbiome, and this balance may help to determine how the microbiome affects a pet’s health (Holmes et al., 2017).

Future research will determine the appropriate balance of saccharolysis and putrefaction to optimize health. A working hypothesis holds that delivery of tryptophan, branched chain amino acids, and adequate saccharolytic substrates into a gut microbiome with the appropriate genetic capacity for transforming these substrates into indole-3-propionate, isobutyrate, and straight SCFAs, will promote optimal health. Examples of the effects of postbiotics on the GI microbiome in several disease states are reviewed below.

### Inflammatory Enteropathies

The postbiotic metabolite, H_2_S, which is derived from sulfur-containing amino acids, is a Janus-faced molecule that is associated with both toxic functions and potential health benefits (Zhang et al., 2013). For example, endogenous production of H_2_S by the host generates an endothelium-derived hyperpolarizing effect to promote vasorelaxation and promotes anti-inflammatory effects (Linden et al., 2008; Martin et al., 2010; Zhang et al., 2013). In contrast, H_2_S generation in the colon by gut microbiota inhibits utilization of the SCFA butyrate by colonocytes through inhibition of mitochondrial metalloproteins and is associated with ulcerative colitis (Roediger et al., 1997). This appears to be an example of dose and site of action determining the outcome of H_2_S generation, such that limiting delivery of sulfur-containing amino acids to the colon by increasing digestibility and reducing dietary levels may provide a benefit to the GI tract.

### Kidney Disease

Certain GI bacteria produce precursors of uremic toxins through fermentation of protein and amino acids. Indoxyl sulfate, p-cresol sulfate, and phenylacetylglutamine have been shown to negatively correlate with kidney function in observational studies (Lin et al., 2011; Barrios et al., 2015; see Table 1). In addition, p-cresyl, its parent compound p-cresol, and indoxyl-sulfate are known to contribute to endothelial damage and oxidative stress (Dou et al., 2004; Lin et al., 2011; Gryp et al., 2017). These toxins are important contributors to the development of kidney disease in cats and dogs (Chen et al., 2018; Summers et al., 2019) but data on the relevance of the effects of specific bacteria on levels of uremic toxins are inconsistent. While many animal studies have shown the benefit of removing indoxyl sulfate by AST-120 (an oral carbonaceous adsorbent used in patients with CKD), no consistent benefit on renal endpoints has been demonstrated in clinical studies with humans (Yamaguchi et al., 2017).

There is a stronger association between the consumption of a high-fiber diet and lower inflammation in populations with CKD, an association that may be related to a reduction in the production and absorption of uremic toxins mediated by microbial metabolism (Krishnamurthy et al., 2012). Prebiotic fibers, such as inulin and pea hull, have been shown to reduce levels of p-cresol, p-cresol sulfate, and blood urea in patients with kidney disease (Meijers et al., 2010; Sirich et al., 2014; Salmean et al., 2015). Digestion-resistant starch also has been shown to reduce indoxyl sulfate and p-cresol sulfate and improve kidney function in rats (Vaziri et al., 2014). These reductions were associated with an increased *Bacteroidetes/Firmicutes* ratio and increases in the genera *Ruminococcus*, *Proteobacteria* and *Sutterella* (Kieffer et al., 2016). Moreover, a study in dogs demonstrated increased production of SCFAs after the consumption of the prebiotics guar gum and sugar beet pulp. These prebiotics are believed to have amino acid sparing effects (Wambacq et al., 2016), an outcome critical to the prevention of cachexia in kidney disease. While this study was conducted in healthy dogs, it suggests the potential for dietary components to modify metabolites relevant to a common comorbidity in kidney disease.

Diets high in fermentable fibers from vegetable and fruit sources resulted in a significant reduction in the advanced glycation end product, pyralline, as well as circulating levels of the postbiotic uremic toxin, 4-ethylphenyl sulfate in dogs and cats (Ephraim-Gebreselassie et al., 2017; Ephraim et al., 2020); 4-ethylphenyl sulfate was previously shown to be increased in an animal model of chronic renal failure (Kikuchi et al., 2010). In dogs fed a diet high in fermentable fibers, decreases in pyralline and 4-ethylphenyl sulfate were associated with an increase in proportions of fecal bacterial populations presumed to be beneficial, including *Ruminococcus*, *Oscillospira*, *Dorea*, and *Slackia*, when compared with dogs consuming a control food (Ephraim-Gebreselassie et al., 2017). Further, in cats, improvement in various markers of kidney health including creatinine, urea, guanidino compounds and homoarginine were also observed in cats consuming a food high in fermentable fibers versus controls (Ephraim-Gebreselassie et al., 2017).

Diets specifically formulated for cats and dogs with kidney disease have been shown to increase the lifespan of pets by controlling signs of uremia (Cline, 2016). The use of ingredients such as fish oil, antioxidants, L-carnitine, and botanicals has resulted in significant improvements in markers of renal health and have also been shown to exhibit beneficial effects on the microbiome (Yu et al., 2014; Fukami et al., 2015; Hall et al., 2016a, b). Although a definitive link between diet, microbiome function, and kidney disease has not been established in cats and dogs, one group reported reductions in several uremic toxins associated with indoles in cats with renal insufficiency after feeding a diet containing a specific combination of a simple fermentable fiber and a complex polysaccharide (Ephraim-Gebreselassie et al., 2018). Findings such as these suggest that further study on the role of nutrition in shaping microbiome function to influence renal health in cats and dogs is needed.

### Allergy and Oral Health

Emerging postbiotics, such as 10-hydroxy-*cis*-12-octadecenoic acid, have generated interest due to their anti-allergic effects and anti-inflammatory activity in the intestine (Miyamoto et al., 2015; Kaikiri et al., 2017; Ikeguchi et al., 2018). A study in mice demonstrated that consumption of 10-hydroxy-*cis*-12-octadecenoic acid reduced clinical symptoms of allergic disease, including scratching behavior, hemorrhage, edema, and dryness (Kaikiri et al., 2017), suggesting it could have similar effects in cats and dogs. This nutritional component, which is found in food-derived polyunsaturated fats, may also be beneficial for other mucosal pathologies, such as oral diseases that stem from decreased epithelial barrier integrity (Yamada et al., 2018). While speculative, emerging postbiotics such as this warrant further research to determine if they can be modulated through nutrition in a way that impacts cat and dog health in a beneficial manner.

## Conclusion and Outlook

The role of the GI microbiome in both health and disease is an increasingly important field of study for researchers and area of clinical interest for veterinarians. The GI microbiome is now recognized as a metabolic organ that plays a critical role in numerous processes essential to the health and fitness of the host. Consequently, disturbances in the microbiome may contribute to or exacerbate illness, while the introduction of nutritional interventions that optimize the composition and function of the microbiome may improve the health of cats and dogs.

The focus of microbiome research has shifted recently from the potential impact of compositional changes to understanding how functional changes achieved through nutrition can enhance overall pet health. This evolving research landscape aligns with the current perspective of the North American branch of the International Life Sciences Institute, which highlighted the need for evidence demonstrating an association between structural changes in the microbiome and function or markers of health (McBurney et al., 2019). Moreover, accumulating evidence suggests that interventions aimed at improving microbiome function may provide significant benefits to the health of cats and dogs. Such an approach will challenge researchers studying pet foods to consider metrics that measure changes in the functional characteristics of the microbiome (Backhed et al., 2012), such as measurements of the metabolites themselves or meta-transcriptomics, in order to develop foods that not only meet the nutritional requirements of cats and dogs, but also enhance the function of their GI microbiome, to support the holistic health of the animal.

Variations in the GI microbiome that have been observed among healthy pets and those with various diseases such as IBD, allergy, oral disease, overweight, diabetes, and kidney disease, suggests that nutritional components that effectively target the microbiome may require tailoring to the unique features of a given health condition. While foods can be formulated with ingredients generally recognized to protect against microbiome disturbances, additional study is needed to demonstrate the impact of such changes on disease in individual cats and dogs. Although use of microbiome-based health screening (Staley et al., 2018) and microbiome diagnostics are not yet widespread, future use of microbial-derived biomarkers for specific diseases may enhance the efficacy and efficiency of diagnosis, assessment of progression, and prognosis (Bäckhed et al., 2005; Davies, 2018), as well as decisions related to the choice of nutrition therapy. Mounting evidence suggests that nutrition influences GI microbiome composition and function, impacts extra-GI organs directly or indirectly, and has reshaped the field of microbiome research in the context of personalized nutrition. Further research is needed to develop consensus around quantifiable measures for characterizing the microbiome in both wellness and disease, evaluating the impact of nutrition interventions and ultimately guiding the development of appropriate nutritional recommendations for microbiome health in cats and dogs.

## Author Contributions

SW, JR, MJ, EE, DB, JM, and DJ wrote the manuscript. SW, JR, MJ, and JS edited and revised the manuscript.

## Conflict of Interest

SW, JR, MJ, EE, DB, and JM were current employees and DJ was a former employee of the Hill’s Pet Nutrition, Inc., Topeka, KS, United States. The remaining author declares that the research was conducted in the absence of any commercial or financial relationships that could be construed as a potential conflict of interest.
